# Changes in subgingival microflora in patients with fixed orthodontic appliances

**DOI:** 10.1590/2177-6709.30.1.e2524154.oar

**Published:** 2025-04-07

**Authors:** Adriana ARBUTINA, Irena Kuzmanović RADMAN, Mirjana UMIĆEVIĆ-DAVIDOVIĆ, Marijana ARAPOVIĆ-SAVIĆ, Aleksandra ĐERI, Radmila ARBUTINA, Saša MARIN, Renata JOSIPOVIĆ, Nataša TRTIĆ

**Affiliations:** 1 University of Banja Luka, Faculty of Medicine, Department of Orthodontics (Banja Luka, Bosnia and Herzegovina).; 2 University of Banja Luka, Faculty of Medicine, Department of Dental Diseases (Banja Luka, Bosnia and Herzegovina).; 3 University of Banja Luka, Faculty of Medicine, Department of Oral Surgery (Banja Luka, Bosnia and Herzegovina).; 4 University of Banja Luka, Faculty of Medicine, Department of Oral Medicine and Periodontology (Banja Luka, Bosnia and Herzegovina).

**Keywords:** Fixed orthodontic therapy, Periodontopathogenic bacteria, PCR, Tratamento ortodôntico fixo, Bactérias periodontopatogênicas, PCR

## Abstract

**Introduction:**

Fixed orthodontic therapy is often accompanied by accumulation of plaque around the orthodontic brackets, which increases the number of periodontopathogenic bacteria.

**Objective:**

The objective of this study was to determine the changes in the subgingival microflora that occurred six months after the placement of a fixed orthodontic appliance.

**Methods:**

The study included 30 patients aged 13 to 35 years, in whom samples of subgingival plaque were taken before and six months after the start of fixed orthodontic therapy from the disto-buccal subgingival space of the left upper (U1) and lower central incisors (L1), mesio-buccal subgingival space of left upper (U6) and lower (L6) first molars. Material samples were tested for the presence of the following bacteria: *Tannarela forsythia, Treponema denticola, Porphyromonas gingivalis, Aggregatibacter actinomycetemcomitans, Prevotella intermedia, Prevotella nigrescens* and *Eikenella corrodens*, using the PCR method.

**Results:**

A significant increase of patients with presence of bacteria especially in the molar region was found: *Tannarela forsythia* (U6 T1 10%-T2 80%, L6 T1 16.67%-T2 80%), *Porphyromonas gingivalis* (U6 T1 60%-T2 90%, L6 T1 60%-T2 83.33%), *Prevotella intermedia* (U6 T1 23.33%-T2 73.33%, L6 T1 26.67%-T2 76.67%), *Prevotella nigrescens* (U6 T1 16.67%-T2 63.33%, L6 T1 23.33%-T2 73.33%) and *Eikenella corrodens* (U6 T1 26.67%-T2 63.33%, L6 T1 23.33%-T2 73.33%) six months after the placement of the fixed orthodontic appliance.

**Conclusion:**

In the initial phase of fixed orthodontic therapy, an increase in the number of patients with periodontopathogenic bacteria *Tannarela forsythia, Treponema denticola, Porphyromonas gingivalis, Prevotella intermedia, Prevotella nigrescens* and *Eikenella corrodens* was observed.

## RESUMO

**Introdução:**

O tratamento ortodôntico fixo é frequentemente acompanhado de acúmulo de placa ao redor dos braquetes, o que aumenta o número de bactérias periodontopatogênicas.

**Objetivo:**

O objetivo desse estudo foi determinar as alterações ocorridas na microflora subgengival seis meses após a colocação de aparelho ortodôntico fixo.

**Métodos:**

O estudo incluiu 30 pacientes com idades entre 13 e 35 anos, dos quais foram coletadas amostras de placa subgengivaln antes do início do tratamento ortodôntico fixo e após seis meses, no espaço subgengival distovestibular dos incisivos centrais esquerdos superior (U1) e inferior (L1), e espaço subgengival mesiovestibular dos primeiros molares esquerdos superior (U6) e inferior (L6). Usando o método PCR, as amostras de materiais foram testadas quanto à presença das seguintes bactérias: *Tannarela forsythia, Treponema denticola, Porphyromonas gingivalis, Aggregatibacter actinomycetemcomitans, Prevotella intermedia, Prevotella nigrescens* e *Eikenella corrodens*.

**Resultados:**

Seis meses após a colocação do aparelho ortodôntico fixo, foi encontrado um aumento significativo de pacientes com presença de bactérias, especialmente na região dos molares: *Tannarela forsythia* (U6 T1 10%-T2 80%, L6 T1 16,67%-T2 80%), *Porphyromonas gingivalis* (U6 T1 60%-T2 90%, L6 T1 60%-T2 83,33%), *Prevotella intermedia* (U6 T1 23,33%-T2 73,33%, L6 T1 26,67%-T2 76,67%), *Prevotella nigrescens* (U6 T1 16,67%-T2 63,33%, L6 T1 23,33%-T2 73,33%) e *Eikenella corrodens* (U6 T1 26,67%-T2 63,33%, L6 T1 23,33%-T2 73,33%).

**Conclusão:**

Na fase inicial do tratamento ortodôntico fixo, foi observado um aumento no número de pacientes com bactérias periodontopatogênicas *Tannarela forsythia, Treponema denticola, Porphyromonas gingivalis, Prevotella intermedia, Prevotella nigrescens* e *Eikenella corrodens*.

## INTRODUCTION

Therapy with fixed orthodontic appliances is one of the most commonly used methods in modern orthodontic practice. One of the complications that can occur during this therapy is gingival inflammation caused by the accelerated accumulation of bacterial plaque around orthodontic brackets. This inflammation is most often accompanied by gingival hyperplasia and bleeding, which slows down and complicates the implementation of the therapy plan. Previous research has shown that the placement of a orthodontic brackets affects subgingival microflora.^1^ Kim et al.^2^ observed an increase of periodonopathogenic bacteria even in the initial leveling stage of orthodontic treatment, especially in the molar region. The accelerated accumulation of dental plaque is influenced by numerous factors such as: the design and size of the orthodontic brackets, the method of ligation, the type of material from which the brackets are made and the level of oral hygiene presented by the patient, as well as the patient’s age.^3^ The presence of supragingival dental plaque in patients with fixed orthodontic appliances can also lead to demineralization and caries. The bacteria *Streptococcus mutans* and *Lactobacilli* are considered to be the main causative agents of these enamel changes. However, *Actinomycetes* bacteria (the so-called “green complex” bacteria) play an important role in the co-aggregation of periodontopathogenic bacteria in subgingival plaque, which consists mainly of gram-negative anaerobic bacteria that lead to changes in the periodontium. The bacteria of the “orange complex” (*Campylobacter rectus, Campylobacter gracilis, Campylobacter showae, Eubacterium nodatum, Fusobacterium nucleatum, Fusobacterium polymorphum, Peptostreptococcus micros, Prevotella nigrescens* and *Streptococcus constellatus*), with their enzymes and toxins, contribute to increasing the depth of the periodontal pockets and create good conditions for the colonization of “red complex” bacteria (*Porphyromonas gingivalis, Prevotella intermedia* and *Tannerella forsythia*) responsible for progressive periodontal damage.^4^

The use of radiographic images during orthodontic therapy and determination of clinical parameters such as probing depth, clinical attachment, bleeding on probing and gingival index cannot provide sufficient data on the periodontal status of patients with fixed orthodontic appliances.^2^ The basic methods used for the detection of microorganisms are cell culturing and polymerase chain reaction (PCR). The PCR method showed greater sensitivity and specificity in the detection of bacteria, especially anaerobes, compared to other methods, which makes it adequate in determining the microbiological status of dental plaque. However, few studies have used PCR to detect bacteria in patients with fixed orthodontic appliances.^5^ ^-^ ^8^

Although numerous studies have shown that there is a change in the subgingival microflora after the placement of a fixed orthodontic appliance, they also found that there is a decrease in the number of bacteria, especially anaerobic ones, after the completion of the therapy. Certain studies have even determined the presence of pathogenic bacteria in the blood of patients after the removal of fixed orthodontic appliances.^9^ ^,^ ^10^

The importance of proper maintenance of oral hygiene in patients with fixed or mobile orthodontic appliance is crucial for the preservation of oral health. The majority of studies mention methods that could improve the oral hygiene of orthodontic patients, such as frequent oral hygiene training and the use of medication to reduce changes in the periodontium.^11^

Therefore, the purpose of the present research was to determine the changes in the subgingival microflora that occurred six months after the placement of a fixed orthodontic appliance.

## MATERIAL AND METHODS

The research was conducted after the approval of the Ethics Committee of University Clinical Centre of Republic of Srpska (01-9-192.2/15). 

### SUBJECTS AND CLINICAL PROCEDURE

Thirty subjects (18 women and 12 men) aged 13 to 35 were included in the research. Subjects were consecutively selected from among patients who arrived for orthodontic treatment at the Department of Orthodontics, Faculty of Medicine, University of Banja Luka. 

Subjects had to meet the following criteria:

» no chronic disease,
» non-smokers,
» not to have large composite fillings, especially proximal fillings near the gingiva,
» no periodontal treatment in the last six months,
» no use of antibiotics and anti-inflammatory drugs three months before the start of the study,
» plaque index and gingival index less than 1.

One month before the placement of the fixed orthodontic appliance, the patients received detailed instructions for maintaining oral hygiene, by a periodontology and oral medicine specialist. In all 30 subjects, subgingival plaque samples were taken before the start of orthodontic therapy using sterile paper points. After ensuring a relatively dry working field using sterile cotton rolls, sterile paper points were placed subgingivally, 1 mm into the gingival sulcus of disto-buccal subgingival space of left upper central incisors (U1), left lower central incisors (L1), mesio-buccal subgingival space of left upper first molars (U6) and left lower first molars (L6). The paper points were left for 30 seconds *in situ* and then they were placed in Eppendorf tubes with 250 ml of distilled water and stored in a freezer at -20°C until PCR analysis.^2^ ^,^ ^12^ After subgingival plaque sampling procedure, the placement of fixed orthodontic appliances was done in patients. The buccal surfaces of the teeth were first treated with 37% orthophosphoric acid Scotchbond™ (3M Unitek, Monrovia, CA, USA) for 20 seconds, rinsed with distilled water for 30 seconds and air dried to frosty-white appearance. A thin layer of primer Transbond^TM^ XT (3M Unitek, Monrovia, CA, USA) was applied to the buccal surfaces of the teeth using an applicator and light-cured for 10 seconds with a LED curing unit (Woodpecker, Guilin, China). A small amount of composite material (Transbond XT; 3M Unitek, Monrovia, Calif) was placed on the bracket’s base (Mini sprint brackets, Forestadent, Pfhorzeim, Germany). Brackets were positioned on the teeth using a suitable instrument, and light-cured for 40 seconds (10 seconds on each side of the bracket). Subgingival plaque samples were taken from each patient six months after the start of orthodontic therapy. Before taking the sample, the ligature elements and the wire were removed, a relatively dry working field was provided, and the samples were taken in the same way as at the beginning of the study.

### POLYMERASE CHAIN REACTION (PCR)

Samples were tested for the presence of the following microorganisms: *T. forsythia, T. denticola, P. gingivalis, A. actinomycetemcomitans, P. intermedia, P. nigrescens* and *E. corrodens*. The samples were tested at the Institute of Human Genetics, Faculty of Dentistry, University of Banja Luka, using the PCR method. Bacterial DNA was isolated by treating the samples with proteinase K (MBI Fermentas, Vilnius, Lithuania) at the temperature of 56°C for 30 minutes, followed by enzyme inactivation by heating the samples at the temperature of 94°C for 15 minutes. Known primer sequences, adequate hybridazation temperatures and expected amplicon lengths were used for the application of PCR analysis, as follows: *Tannarela forsythia* - GTA GAG CTT ACA CTA TAT CGC AAA CTC CTA (53°C, 600bp), *Treponema denticola* - TAA TAC CGA ATG TGC TCA TTT ACA T TCA AAG AAG CAT TCC CTC TTC TTC TTA (60°C, 316bp), *Porphyromonas gingivalis* - CAA TAC TCG TAT CGC CCG TTA TTC (55°C, 400bp), *Aggregatibacter actinomycetemcomitans* - CAC TTA AAG GTC CGC CTA CGT GC (55°C, 500bp), *Prevotella intermedia* - GTT GCG TGC ACT CAA GTC CGC C (53°C, 660bp), *Prevotella nigrescens* - CGTTGGCCCTGCCTGCGG (55°C, 804bp), *Eikenella corrodens* - CTA ATA CCG CAT ACG TCC TAA G CTA CTA AGC AAT CAA GTT GCC C (55°C, 800bp). Positive controls included the DNA obtained from reference strains of microorganisms, and negative controls included distilled water without DNA sample.^12^ ^,^ ^13^

PCR was made in a volume of 25µl, and the reaction mixture had the following content: sterile water, PCR buffer, 2.5mM MgCl_2_, 200µM dNTP, 5µM primers, 0.1 U Taq polymerase, 4µl sample with assumed bacterial DNA. Performance temperature profile of PCR analysis was: initial denaturation at 95°C for 3 minutes, followed by a cycle of 35 repetitions: denaturation at 94°C for 60 seconds, hybridization at 55°C for 60 seconds, elongation 72°C for 60-90 seconds and final elongation 72°C for 7 minutes. Using 8% polyacrylamide gel electrophoresis, staining with ethidium bromide, and finally visualization after exposure to ultraviolet (UV) light, the PCR products were analyzed. 

This study was designed in order to estimate a possible change in the bacterial DNA presence in the subgingival biofilm, in relation to early phase of orthodontic therapy with fixed orthodontic appliance.

### STATISTICAL ANALYSIS

The statistical package IBM SPSS Statistics for Windows, version 20.0. (IBM Corp. Armonk, NY, USA) and Microsoft Excel 2010 (Windows, Redmond, WA, USA) were used for data analysis. The number of patients with presence of bacteria in subgingival plaque was expressed as frequencies and percentage. The dependent t-test for paired samples was used to compare the mean values ​​of the number of patients with the presence of bacteria before and six months after the placement of the fixed orthodontic appliance. Values of p<0.05 were taken as statistically significant.

## RESULTS

A significant increase of patients with periodontopathogens was observed after six months of orthodontic brackets placement: *T. forsythia* (U1 - T1 6.66%-T2 23.33%, U6 - T1 10%-T2 80%, L1 - T1 16.67%-T2 43.33%, L6 - T1 16.67%-T2 80%), *P. gingivalis* (U1 - T1 30%-T2 36.67%%, U6 - T1 60%-T2 90%, L1 - T1 23.33%-T2 53.33%, L6 - T1 60%-T2 83.33%), *P. intermedia* (U1 - T1 20%-T2 40%, U6 - T1 23.33%-T2 73.33%, L1 - T1 10%-T2 46.67%, L6 - T1 26.67%-T2 76.67%), *P. nigrescens* (U1 - T1 16.67%-T2 36.67%, U6 - T1 16.67%-T2 63.33%, L1 - T1 16.67%-T2 43.33%, L6 - T1 23.33%-T2 73.33%) and *E. corrodens* (U1 - T1 33.33%-T2 36.67%, U6 - T1 26.67%-T2 63.33%, L1 - T1 20%-T2 30%, L6 - T1 40%-T2 70%). Table 1 shows the number of patients with the presence of periodontopathogens in subgingival plaque before and six month after the placement of a fixed orthodontic appliance. In the samples taken from the subgingival plaque of the upper and lower first molar, a higher presence of bacteria was observed, compared to the incisors (Table 1).

**Table 1:** Number of patients with the presence of periodontopathogens in subgingival plaque before (T1) and 6 months after placement of the fixed orthodontic appliance (T2).

Bacteria	Upper left central incisor		Upper left first molar		Lower left central incisor		Lower left first molar	
	n (%)		n (%)		n (%)		n (%)	
	T1	T2	T1	T2	T1	T2	T1	T2
Tf	2 (6.66%)	7 (23.33%)	3 -10%	24 -80%	5 (16.67%)	13 (43.33%)	5 (16.67%)	24 -80%
Td	6 -20%	6 -20%	19 (63.33%)	23 (76.67%)	17 (56.67%)	12 -40%	18 -60%	25 (83.33%)
Pg	9 30%)	11 (36.67%)	18 -60%	27 -90%	7 (23.33%)	16 (53.33%)	18 -60%	25 (83.33%)
Aa	12 -40%	10 (33.33%)	27 -90%	23 (76.67%)	14 (46.67%)	19 (63.33%)	26 (86.67%)	25 (83.33%)
Pi	6 -20%	12 -40%	7 (23.33%)	22 (73.33%)	3 -10%	14 (46.67%)	8 (26.67%)	23 (76.67%)
Ec	10 (33.33%)	11 (36.67%)	8 26.67%)	19 (63.33%)	6 -20%	9 -30%	12 -40%	21 -70%
Pn	5 (16.67%)	11 (36.67%)	5 (16.67%)	19 (63.33%)	5 (16.67%)	13 (43.33%)	7 (23.33%)	22 (73.33%)

Tf = *Tannarela forsythia,* Td = *Treponema denticola,* Pg = *Porphyromonas gingivalis,* Aa =*Aggregatibacter actinomycetemcomitans,* Pi = *Prevotella intermedia,* Ec = *Eikenella corrodens,* Pn =*Prevotella nigrescens.*

**Figure 1:**
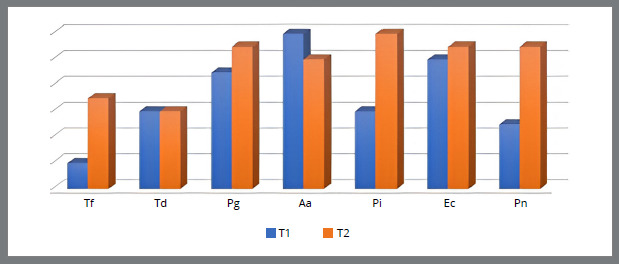
Number of patients with the presence of bacteria in the subgingival plaque of the upper left central incisor before placement (T1) and six months after placement of the fixed orthodontic appliance (T2).

**Figure 2: f2:**
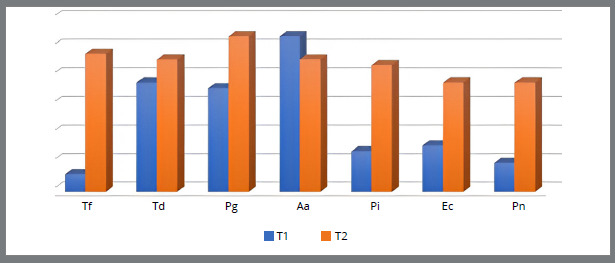
Number of patients with the presence of bacteria in the subgingival plaque of the upper left first molar before placement (T1) and six months after placement of fixed orthodontic appliance (T2).

**Figure 3: f3:**
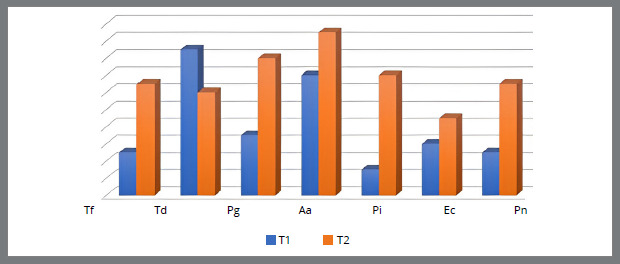
Number of patients with the presence of bacteria in the subgingival plaque of the lower left central incisor before placement (T1) and six months after placement of the fixed orthodontic appliance (T2).

**Figure 4:**
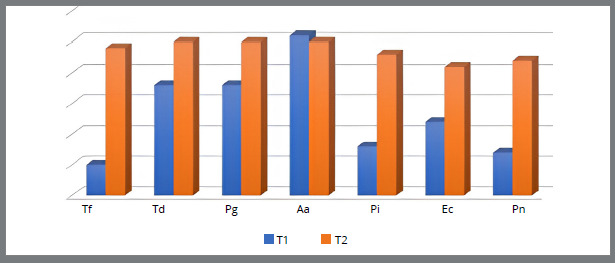
Number of patients with the presence of bacteria in the subgingival plaque of the lower left first molar before placement (T1) and six months after placement of fixed orthodontic appliance (T2).

Figures 1 to [Fig f2] [Fig f3] 4 show the relationship between the number of patients with the presence of bacteria in the subgingival plaque of the upper left central incisor and upper left first molar, and the lower left central incisor and lower left first molar before placement and after six months of fixed orthodontic appliance therapy. It is observed that the number of patients with periodontopathogens is significantly higher after six months of the orthodontic treatment, except for the *A. actinomycetemcomitans* (U1, U6, L6) (Fig. 1-4).

Using the dependent t-test for paired samples, a statistically significant difference was found in the number of patients with the presence of all tested bacteria before and after the placement of the fixed orthodontic appliance in upper left first molar, lower left central incisor and lower left first molar; while no statistically significant difference was found in the upper left central incisor (*p* = 0.07443) (Table 2).

**Table 2:** Comparison of the number of patients with the presence of all tested bacteria in subgingival plaque of the examined teeth before and six months after placement of a fixed orthodontic appliance.

		T1/T2
Upper left central incisor (U1)	t	-2.15655
	p	0.07443
Upper left first molar (U6)	t	-3.25669
	p	0.01732
Lower left central incisor (L1)	t	-2.75542
	p	0.033054
Lower left first molar (L6)	t	-3.99406
	p	0.007167

## DISCUSSION

During therapy with a fixed orthodontic appliance, due to inadequate maintenance of oral hygiene and increased accumulation of dental plaque, gingivitis and an increase in the depth of periodontal pockets may occur. In rare cases, gingival recession, appearance of gingival black triangles and loss of interdental papilla, resorption of tooth roots and bone dehiscence can also occur, but these phenomena are more related to inadequately planned orthodontic therapy.^14^ ^,^ ^15^ Previous research has shown that during orthodontic therapy, there is a change in the microbiological status of dental plaque, and an increase in the number of periodontopathogenic bacteria.^16^ ^-^ ^18^

In this study, only the presence or absence of bacteria in subgingival plaque was examined in patients before and six months after the placement of a fixed orthodontic appliance. All patients were trained to maintain proper oral hygiene before the start of orthodontic therapy, however, there was a significant increase in the presence of bacteria in patients six months after the start of therapy. Although quantification of bacteria was not done, this study investigated the presence of seven different species that are most often responsible for changes in the periodontium in patients in general. For all bacteria, an increased presence was observed in the tested samples, except for the *A. actinomycetemcomitans*. All patients were treated with the same type of metal brackets (not nano-coated), which are used in the labial technique.^19^ *A. actinomycetemcomitans* is a gram-negative bacterium that is associated with the early stages of periodontal diseases, and whose increase is directly related to the increase in the depth of periodontal pockets.^20^ Lemos et al.^21^, while examining the impact of fixed orthodontic therapy on the periodontal health of patients, determined that there was no significant increase in the number of bacteria *A. actinomycetemcomitans*. Previous research has shown that in patients with fixed orthodontic appliances, changes in the microbial flora occur during the first weeks of treatment. The primary colonizers were found to be *Streptococci* and *Actynomycetes* and, after one month of orthodontic treatment, orange complex bacteria levels increased in the subgingival bacterial population.^22^ This could explain why significant increase in the presence of *A. actinomycetemcomitans* was not observed in the examined samples.

Other authors concluded a significant increase of bacteria: *T. denticola, P. gingivalis* and *T. forsythia* during the first three months of orthodontic treatment, which is in accordance with results of the present study.^1^ ^,^ ^2^ Similarly, Hussain et al.^23^ observed a significant increase of *T. denticola* in patients with fixed orthodontic appliances and in a group of patients who did not have gingival enlargement caused by dental plaque. Ravi et al.^24^ and Vijayan et al.^25^ also observed an increase in the number of red complex bacteria (*T. denticola, P. gingivalis* and *T. forsythia*) after three and after six months of fixed orthodontic appliance placement. In addition to changes in the subgingival microflora, they observed pathological changes in the periodontium, and found an increase in the plaque index, bleeding on probing, and an increase in the depth of periodontal pockets. In the tested samples taken from the molar region of patients with the fixed orthodontic appliances, a significantly higher presence of bacteria *E. corrodens* was observed six months after the start of orthodontic therapy. The very presence of this bacterium was investigated because it is often associated with the appearance of gingivitis in adults. Some authors pointed out that sometimes the increase of this endogenous pathogen contributes to the appearance of periodontitis.^26^

Although all patients were trained in adequate maintenance of oral hygiene before the start of the study, it was observed that the number of patients with presence of bacteria found on samples from the subgingival surfaces of the first permanent molars was higher than the number of patients with the presence of same bacteria from samples taken from the central incisors. This result could perhaps be explained by easier access to the incisors and the spaces around the incisor brackets during maintaining oral hygiene, compared to the molars.

However, Cenzato et al.^27^ indicate the importance of maintaining good oral hygiene in patients with fixed orthodontic appliances, not because brackets are more susceptible to plaque accumulation, but because plaque bacteria are more pathogenic in these patients. Also, Cota-Quintero et al.^28^ concluded that in patients treated with conventional fixed orthodontic appliances and self-ligating appliances, there is an increase in the number of periodontogenopathogenic bacteria *T. denticola, P. intermedia and Fusobacterim nucleatum*, and that the longer the therapy lasts, the greater are the changes in the subgingival microflora.

Some studies have compared changes in the subgingival microflora in patients with different types of orthodontic appliances. In the present research, preference was given to the labial fixed appliance, considering the most common application in adult population. Examining the changes in the oral microflora in patients with lingual brackets, labial brackets and orthodontic aligners, Gujar et al.^29^ concluded that there was an increase in a certain number of bacteria to a lesser or greater extent depending on the type of therapy used. In patients with labial fixed appliances, a statistically significant increase in *P. intermedia* bacteria was observed, although an increase in other tested bacteria was also observed, which is in line with the results of this study.^29^ Wang et al.^30^ found a significant increase in the number of bacteria associated with periodontal diseases, and an increase in parameters that favor them, such as the gingival and plaque index, in patients with fixed orthodontic appliances compared to patients with orthodontic aligners. Also, changes in the oral microflora are associated with additional intraoral elements that are used during the orthodontic therapy itself. A limitation of this study is the examination of the change in subgingival microflora only in patients with fixed orthodontic appliances, small sample size and the examination of only presence or absence of bacteria in subgingival plaque before and six months after orthodontic appliance placement. But the number of examined bacteria is significant and very important for better understanding the changes in subgingival microflora that occur during therapy with fixed orthodontic appliance.

## CONCLUSION

Six months after placement of the fixed orthodontic appliance, an increase in the number of patients with the presence of bacteria *T. forsythia, T. denticola, P. gingivalis, P. intermedia, P. nigrescens* and *E. corrodens* was observed. Proper maintenance of oral hygiene during orthodontic therapy, as well as in the retention period after completion of orthodontic therapy, is necessary in order to carry out the planned therapy successfully to the end. Although patients are trained in proper oral hygiene before the start of therapy, it is necessary to perform additional preventive measures and regularly monitor the condition of the gingiva and the level of oral hygiene maintenance, in order to prevent changes in the periodontium.
